# The role of surgery following incomplete response to high-dose IL-2 (HD IL-2)

**DOI:** 10.1186/2051-1426-1-S1-P145

**Published:** 2013-11-07

**Authors:** Tasha Hughes, Gail Iodice, Sanjib Basu, Steven Bines, Howard Kaufman

**Affiliations:** 1 Rush University Medical Center, Chicago, IL, USA; 2 Columbia University Medical Center, New York, NY, USA

## Background

Surgical resection of metastatic cancer is beneficial in select patients with cancer. HD IL-2 is an FDA approved immunotherapy for the treatment of patients with melanoma or renal cell carcinoma. IL-2 induces a complete response (CR) in 4-10% of patients while an additional 10% of patients have a partial response (PR). While not frequently reported, our experience suggests an additional 20% of patients have a stable response (SD) to therapy. The subsequent management of patients with an incomplete response, either partial or stable, has not been well studied and we sought to determine if metastasectomy might have a role in this setting.

## Methods

305 patients with metastatic renal cell carcinoma or melanoma treated with IL-2 therapy over a 12-year period were reviewed. Age, response and survival data were available for 215 patients. Response was determined using standard RECIST criteria and patients with partial response (PR) or stable disease (SD) were considered incomplete responders to IL-2. Patients with an incomplete response were evaluated by a surgical oncologist. Surgical complete response (sCR) was defined as complete surgical resection of a single or multiple sites of disease following IL-2 therapy that rendered patients free of disease. Overall survival was estimated analyzed using Kaplan-Meier curves and compared between groups using the log-rank test.

## Results

The objective response rate (PR + CR) to HD IL-2 in this cohort was 13.6%. An additional 24.4% of patients had SD following their initial course of therapy. Median survival of all treated patients was 16.8 months. Incomplete response to IL-2 does confer an improvement in overall survival compared to patients with progressive disease (median survival 38.2 v. 7.9 months). Eighty-one patients had an incomplete response (PR + SD) to IL-2, fifteen of whom underwent subsequent metastasectomy. Patients undergoing metastasectomy had improved overall survival compared to patients with an incomplete response that did not undergo subsequent surgery (38.2 months v. median not reached in surgical patients, p=0.026). Of patients treated surgically following HD IL-2 12 patients were alive at the end of follow-up with follow-up ranging from 15 months to 96 months.

## Conclusion

The addition of surgical resection may improve upon the survival benefit in select patients with incomplete response to HD IL-2. These findings are biased by patient selection, but our results support the rationale for a prospective trial to determine the role of metastasectomy following incomplete response to IL-2 therapy. Additionally, we are interested in understanding how surgical resection following immunotherapy may reset immunologic balance in patients with metastatic cancer.

